# Prevalence of influenza A viruses in livestock and free-living waterfowl in Uganda

**DOI:** 10.1186/1746-6148-10-50

**Published:** 2014-02-27

**Authors:** Halid Kirunda, Bernard Erima, Agnes Tumushabe, Jocelyn Kiconco, Titus Tugume, Sophia Mulei, Derrick Mimbe, Edison Mworozi, Josephine Bwogi, Lukwago Luswa, Hannah Kibuuka, Monica Millard, Achilles Byaruhanga, Mariette F Ducatez, Scott Krauss, Richard J Webby, Robert G Webster, Kofi Wurapa, Denis K Byarugaba, Fred Wabwire-Mangen

**Affiliations:** 1 National Livestock Resources Research Institute, P.O. Box 96, Tororo, Uganda; 2 Makerere University Walter Reed Project, P.O. Box 16524, Kampala, Uganda; 3 Mulago National Referral Hospital, Department of Paediatrics and Child Health/Makerere University College of Health Sciences, P.O. Box 7072, Kampala, Uganda; 4 Uganda Virus Research Institute, P.O. Box 49, Entebbe, Uganda; 5 Epidemiology and Surveillance Division, Ministry of Health, P.O. Box 7272, Kampala, Uganda; 6 NatureUganda, The East Africa Natural History Society, P.O. Box 27034, Kampala, Uganda; 7 INRA UMR1225 IHAP Interactions hôtes-agents pathogènes, ENVT, Toulouse, France; 8 Department of Infectious Diseases, St. Jude Children's Research Hospital, Memphis, USA; 9 U.S. Army Medical Research Unit-Kenya, U.S. Embassy, P.O. Box 606, Nairobi, Kenya; 10 College of Veterinary Medicine, Animal Resources and Biosecurity, Makerere University, P.O. Box 7062, Kampala, Uganda; 11 School of Public Health, College of Health Sciences, Makerere University, P.O. Box 7072, Kampala, Uganda

**Keywords:** Influenza A viruses, Poultry, Pigs, RNA, Sera, Free-living, Waterfowl

## Abstract

**Background:**

Avian influenza viruses may cause severe disease in a variety of domestic animal species worldwide, with high mortality in chickens and turkeys. To reduce the information gap about prevalence of these viruses in animals in Uganda, this study was undertaken.

**Results:**

Influenza A virus prevalence by RT-PCR was 1.1% (45/4,052) while seroprevalence by ELISA was 0.8% (24/2,970). Virus prevalence was highest in domestic ducks (2.7%, 17/629) and turkeys (2.6%, 2/76), followed by free-living waterfowl (1.3%, 12/929) and swine (1.4%, 7/511). A lower proportion of chicken samples (0.4%, 7/1,865) tested positive. No influenza A virus was isolated. A seasonal prevalence of these viruses in waterfowl was 0.7% (4/561) for the dry and 2.2% (8/368) for the wet season. In poultry, prevalence was 0.2% (2/863) for the dry and 1.4% (24/1,713) for the wet season, while that of swine was 0.0% (0/159) and 2.0% (7/352) in the two seasons, respectively. Of the 45 RT-PCR positive samples, 13 (28.9%) of them were H5 but none was H7. The 19 swine sera positive for influenza antibodies by ELISA were positive for H1 antibodies by HAI assay, but the subtype(s) of ELISA positive poultry sera could not be determined. Antibodies in the poultry sera could have been those against subtypes not included in the HAI test panel.

**Conclusions:**

The study has demonstrated occurrence of influenza A viruses in animals in Uganda. The results suggest that increase in volumes of migratory waterfowl in the country could be associated with increased prevalence of these viruses in free-living waterfowl and poultry.

## Background

Avian influenza (AI) viruses may cause natural infection in a variety of domestic and wild bird species throughout the world and have particularly been reported to occur in poultry either in the highly pathogenic or low pathogenic forms [1]. In addition to the sixteen previously known haemagglutinin (H1–H16) and nine neuraminidase (N1–N9) subtypes identified from avian species, H17N10 and H18N11 subtypes have recently been detected in bats [2] representing the entire pool of influenza A viruses known today. AI viruses have been isolated from at least 105 wild bird species of 26 different families [3]. Among all species where influenza viruses have been isolated, waterfowl and shorebirds are regarded the main reservoirs of these viruses [3]. The prevalence of these viruses has been reported in waterfowl [3], domestic birds and pigs in several parts of Africa [4,5].

Uganda is a seasonal shelter of about 240,000 of the estimated 50 billion birds that make predictable seasonal movements between the temperate zone and the tropics [6] including about 5.4 million ducks [7]. Due to the favourable climate and numerous fresh waterbodies, Uganda serves as a migratory destination for both Palaearctic and intra-Africa migratory species of birds with many wintering in the area for several months [8]. Among the regularly observed birds are eight bird species which are regarded high risk species in the spread of influenza viruses around the world [9,10]. These include Tufted duck (*Aythya fuligula*), long-tailed Cormorant (*Phalacrocorax africanus*) Great Cormorant (*Phalacrocorax carbo*), Northern Shoveler (*Anas clypeata*), Garganey (*Anas querquedula*), Black-headed Gull (*Chroicocephalus ridibundus*) and Eurasian Wigeon (*Anas penelope*). Waterfowl can be a source of low pathogenic avian influenza (LPAI) viruses for domestic avian populations [11], in which they can evolve into highly pathogenic avian influenza (HPAI) strains [12]. Spread of these viruses to domestic species can be favoured by the large number of non-wetland dependant groups (“bridge species” such as cattle and little egrets) that move between free-living waterfowl and human settlements and may interact with domestic birds [11]. The other factors that may facilitate spread of these viruses are the numerous live bird markets (LBMs) scattered across the country. Outbreaks due to HPAI subtypes cause death in poultry and sometimes also in humans. This study was undertaken to establish evidence of exposure of poultry, pigs and wild birds in Uganda to influenza A viruses.

## Methods

### Study area

The study was conducted in 25 districts in central, eastern, northern and western regions of Uganda. Sampling sites included 34 live birds markets (LBMs), 9 sites at lake shores and 9 waterfowl roosting sites. The selected markets were among those where risky environment, hygiene and management practices were observed in a study (unpublished results) previously undertaken to assess biosecurity in LBMs in the country. Such markets and the sites at the shores of Lake Victoria constituted the clusters in the sampling frame. The other study sites comprised 10 waterbodies that regularly provide habitat for at least four waterfowl species previously associated with the spread of influenza viruses [9]. These sites were located in 10 districts.

### Sampling

Samples were collected from farm animals including chickens, turkeys, domestic ducks, guinea fowl and pigs in LBMs, as well as free-living waterfowl. The study used purposive sampling method. During each sampling visit, each poultry trader in a LBM provided two birds collected from different areas until twenty birds were obtained. In addition, pigs brought to the markets from different villages or households were equally used for sample collection. In situations where pigs available in LBMs were not enough for the required number of samples, pigs in farm households within a distance of 1.5 km from the study market were selected to complete the remaining number. Only 2 - 3 pigs were randomly sampled per farm household to complete the required number of swine samples. Since no outbreak of influenza had been reported in the country, a series of three sample collection periods each lasting three months was undertaken. This interval was used in both farm animals and migratory waterfowl except for two sites known to host the largest population of birds throughout the year, where sample collection was done on monthly basis. The total study period was 18 months.

### Sample size

To determine the sample size, the number of sampling elements per cluster (number of birds in a LBM on a sampling visit) was fixed as 30 samples. Based on the fixed number of sampling elements, the number of clusters was determined using the formula for determining the number of clusters for a 95% confidence interval provided by Thrusfield [13]. Since in a recent study [14], the prevalence of influenza virus in migratory waterfowls was 3.5%, a prevalence of about 1% was estimated for a country regularly visited by migratory waterfowls and in which poultry are on a free-range management system. Since no data on variance between clusters existed and no single cluster had had any evidence of infection or exposure, variance of rare diseases [13] was assumed. The variance between clusters was then taken to be 0.01. With fixed sampling elements of 30 per cluster and precision of 0.05, at least 31 clusters were required for sampling during the study. Hence, at least 30 and 10 sera and; 30 and 10 swabs for poultry and pigs, respectively were required from each site during each period of three months of dry season or wet season. Each animal was sampled once during the study period. A total of 4,016 samples were collected for detection of influenza A virus. These included 1,248 cloacal and 1,328 cloaco-oropharyngeal swabs from poultry, while 511 nasal swabs and 929 fresh faecal samples were from swine and waterfowl, respectively. The cloaco-oropharyngeal samples were two separate swabs, both taken from the same bird and combined. Other samples comprised blood for sera from 2,572 poultry and 417 pigs. Table 1 shows the distribution of the different samples among livestock species and regions of sample collection.

**Table 1:** Distribution of sampled poultry and swine in different regions of Uganda based on different parameters

**Region**	**Species**	**Sex**		**Age**			**Breed**			**Mgt system**		**Health status**		**Season**	
		**Female**	**Male**	**Juvenile**	**Grower**	**Adult**	**Local**	**Exotic**	**Ext.**	**Semi**	**Int.**	**Health**	**Sick**	**Dry**	**Wet**
Central (*N = 388*)	Chicken	198	162	-	36	324	209	151	221	-	139	349	11	96	264
	Dom. duck	18	5	-	8	15	23	-	23	-	-	23	-	14	9
	Turkey	1	2	-	-	3	3	-	3	-	-	3	-	-	3
	Guinea fowl	-	2	-	-	2	2	-	2	-	-	2	-	-	2
Eastern (*N = 976*)	Chicken	258	164	2	71	349	422	-	422	-	-	400	22	160	262
	Dom. duck	329	211	24	180	336	540	-	540	-	-	540	-	108	432
	Turkey	1	9	-	-	10	10	-	10	-	-	9	1	4	6
	Guinea fowl	3	1	-	-	4	4	-	4	-	-	4	-	4	-
Northern (*N = 767*)	Chicken	318	401	3	196	520	671	48	691	-	28	719	-	302	417
	Dom. duck	7	7	-	5	9	14	-	14	-	-	14	-	6	8
	Turkey	9	23	-	4	28	32	-	32	-	-	32	-	3	29
	Guinea fowl	2	-	-	-	2	2	-	2	-	-	2	-	-	2
Western (*N = 445*)	Chicken	213	149	2	67	304	333	40	316	-	57	373	-	139	234
	Dom. duck	22	30	-	3	38	41	-	41	-	-	41	-	12	29
	Turkey	8	23	-	4	27	31	-	31	-	-	31	-	15	16
	Guinea fowl	-	-	-	-	-	-	-	-	-	-	-	-	-	-
***Total:***		***1,387***	***1,189***	***31***	***574***	***1,971***	***2,337***	***239***	***2,352***	***-***	***224***	***2,542***	***34***	***863***	***1,713***
Central	Swine	17	3	-	11	9	-	20	-	-	20	20	-	-	20
Eastern	Swine	97	42	-	60	79	105	34	91	31	17	139	-	50	89
Northern	Swine	139	67	30	97	79	133	73	119	10	77	206	-	69	137
Western	Swine	86	60	42	78	26	47	99	79	-	67	146	-	40	106
***Total :***		***339***	***172***	***72***	***246***	***193***	***285***	***226***	***289***	***41***	***181***	***511***	***-***	***159***	***352***

Key: Mgt System = Management system; Ext. = Extensive/Free-range; Semi = Semi-intensive; Int. = Intensive; Dom. ducks = Domestic ducks; Season = Wet season (months of March - May and October – December) or Dry season (January – February and June – September).

### Sample collection and preservation

Dacron tipped swabs were used for faecal, nasal, cloacal and cloaco-oropharyngeal sample collection in virus transport medium. In free-living waterfowl, fresh faecal samples were collected from the environment. Samples were kept on ice during collection and immediately preserved in a nitrogen dry shipper for shipment to the laboratory. In the laboratory, samples were preserved at -70°C until further processing. For sera, 2 ml of blood were collected from each bird and 5 ml from each pig using a procedure described by FAO/APHCA [15] and Framstad and others [16], respectively, and sera were separated and stored at -20°C until used. During sample collection, factors about the sample bird were recorded. These included sex, age, breed, management system, health status and season. Health status was regarded healthy or sick. A bird was considered sick if it had at least three of the clinical signs including respiratory signs (sneezing, gasping, coughing), discharge from eyes, nares or beak, swollen face, combs and/or wattles, bluish combs and wattles and diarrhea. Others were hemorrhages on skin of shanks and breast, twisted necks and paralyzed wings or legs.

### Laboratory analysis

#### RNA extraction

RNA extraction from faecal samples and cloacal, cloaco-oropharyngeal and nasal swabs was done using the QIAamp® Viral RNA Mini extraction kit as described by the manufacturer in the QIAamp®Viral RNA Mini Handbook (Qiagen).

### PCR sample analysis

Detection of influenza A virus was done using primers targeting matrix gene in a single step RT-PCR. The primers with the following sequences were sourced from TAG Copenhagen A/S, Symbion, Fruebjergvej3, DK-2100 Copenhagen (http://tagc.dk/) and used were previously described (17) and were:

Forward primer: 5’-CTTCTAACCGAGGTCGAAAACG-3`[17]; Reverse primer M253R: 5’-AGGGCATTTTGGACAAG/TCGTCTA-3’ [17]; Matrix 3 probe: 5’-Fam-TCAGGCCCCCTCAAAGC-BHQ-1-3’ [18].

Reverse transcription and cDNA amplification were done by real-time reverse transcriptase polymerase chain reaction (rtRT-PCR) using Applied Biosystem 7500 Fast Real-Time PCR System at threshold cycle (Ct) – value of 37.

RNA of samples that were positive by rtRT-PCR was also tested using conventional RT-PCR (cRT-PCR). This was conducted using Veriti 96 Well Thermal Cycler Applied Biosystem. The following primers were used in each reaction:

Forward primer: 5’-TAACCGAGGTCGAAACGTA-3’; Reverse primer M253R: 5’-AGGGCATTTTGGACAAG/TCGTCTA-3.

All oligonucleotide primers were from TAG Copenhagen A/S, Symbion, Fruebjergvej3, DK-2100 Copenhagen (http://tagc.dk/). All samples were run with two positive and two negative controls.

Samples that were positive by the two methods were sub-typed by cRT-PCR using specific oligonucleotide primers for H5 and H7 influenza virus subtypes. PCR positive samples were cultured for virus isolation in 9 – 10 day-old embryonated chicken eggs for three days in three passages before deducing that they were negative by haemagglutination test. Egg inoculation and virus isolation procedures were performed as described [19]. All laboratory procedures were handled under biosafety level 2+ (BSL-2+) laboratory, Influenza Research Laboratory at the College of Veterinary Medicine, Animal Resources and Biosecurity of Makerere University.

### ELISA sample analysis

All sera were analysed for antibodies against influenza A viruses using a multispecies ELISA Kt, IDEXX Influenza A Ab Test With Confidence™ (IDEXX Laboratories, Inc., One IDEXX Drive, Westbrook, Maine 04092, USA), according to manufacturer’s instructions. ELISA reading was done using Biotek Elx 800 96 well ELISA Microplate Reader.

### Haemagglutination inhibition analysis

All ELISA-positive samples were re-analysed using Haemagglutination inhibition (HAI) test for subtyping for H1, H3, H5, H6, H7 and H9. Sera of both poultry and swine were treated with receptor destroying enzyme (RDE) prior to use in HAI. All procedures involving sera separation, RDE treatment and HAI were conducted as described [20]. Titres were only considered positive at ≥ Log_2_4.

### Data management and analysis

Generated data were entered and stored in *EpiInfo* and analysed by *SPSS 16.0* statistical programs. Quantitative data were analysed by descriptive statistics and figures drawn in *Microsoft Excel.* Relationships between independent variables (species, sex and age group) and results of each of RT-PCR and ELISA test results (dependent variables) was done using logistic regression analysis. Strength of existing relationship was determined by computing the chi-square (*χ*^2^), odds ratio (OR) and confidence interval (CI), significant at <0.05.

### Ethical considerations

The study was approved by the Ethics Committee of the College of Veterinary Medicine, Animal Resources and Biosecurity of Makerere University. Permission to conduct the study was granted by Uganda National Council for Science and Technology (Ref. NS 345).

## Results

### Bird species and factors at sites of sample collection

During the study period (2010-2011), it was observed that some of the commonly known influenza A virus spreading free-living waterfowl species were sighted at sites of sample collection. These birds were mainly sighted in March, April, June, September and November. Comparatively, it was only during the same months that positive samples were detected in the study. The sighted risky waterfowl included Long-tailed Cormorant (*Phalacrocorax africanus*), Great Cormorant (*Phalacrocorax carbo*), Egyptian Goose (*Alopochen aegyptiacus*) and Garganey (*Anas querquedula*). Others were Black-headed Gull (*Chroicocephalus ridibundus*), Yellow-billed Duck (*Anas undulata*), White-winged Tern (*Chlidonias leucopterus*) and Gull-billed Tern (*Sterna nilotica*)*.* While some sites had less than 300 individual birds, as many as 500 - 12,146 birds were occasionally counted at the sites during sample collection. Among the 929 fresh faecal swabs taken from roosting sites of these birds, most of them were from marshy soils (31.9%, n = 296), rocks (24.1%, n = 224) and sandy soils (16.6%, n = 155). Site conditions for collection of the remaining faecal (environmental) samples from these birds were dry clay (13.4%, n = 125), degraded soils (7.3%, n = 68) and flooded (6.7%, n = 61) site conditions.

### Spatial variation in occurrence of influenza A viruses

The study observed that influenza A viruses circulated in wild and farmed birds, as well as swine in the four regions of Uganda. Influenza A viruses and antibodies against these viruses were detected in 12 out of the 25 study districts. Among these, only six had both PCR and ELISA positive samples. Regionally, positive samples were from one out of the three districts in central, three out of nine in eastern, two of the seven in northern and two of the six sampled districts in western region. No influenza A virus was detected in all the 165 and 131 samples collected from migratory waterfowl in Eastern and Western region, respectively. Nevertheless, it was detected in 1.9% (12/633) samples from central Uganda. In Table 2, proportions of PCR and ELISA-positive samples distributed in the different regions are shown. Distribution of positive sera among the different regions revealed that the highest proportion of the positive samples were from the western (1.4%, 8/585) followed by northern region (1.0%, 10/970). Eastern and central regions had lower proportions (Table 2).

**Table 2:** Positive PCR and ELISA samples taken from domestic animals by region

**Region of sample collection**	**Swab samples collected**	**PCR positive samples**		**Sera samples collected**	**ELISA positive sera**	
		**Number**	**Percent**		**Number**	**Percent**
Central	388	12	3.1	386	2	0.5
Eastern	976	15	1.5	976	4	0.4
Northern	767	2	0.3	766	10	1.3
Western	445	16	3.6	444	8	1.8
**Total/Average**	**2,576**	**45**	**1.7**	**2,752**	**24**	**0.8**

### Temporal variation in occurrence of influenza A viruses among different species

**Figure 1:**
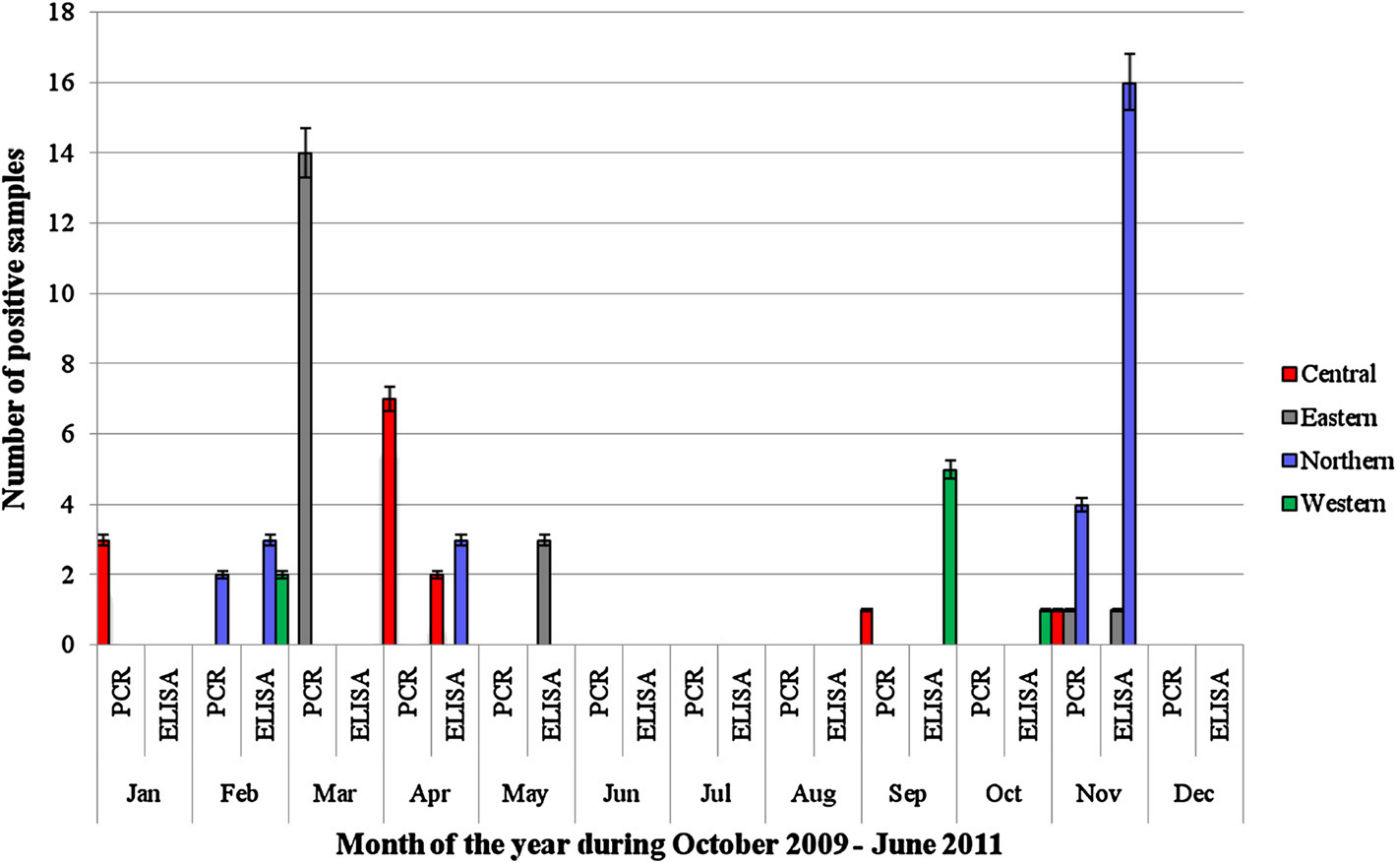
**Temporal distribution of RT-PCR and ELISA positive samples.** Key: Fig = Figure; RT-PCR = Reverse transcriptase polymerase chain reaction; ELISA = Enzyme-linked immunosorbent assay; Jan = January; Feb = February, Mar = March; Apr = April; May = May; Jun = June; Jul = July; Aug = August; Sep = September; Oct = October; Nov = November; Dec = December.

The study observed that exposure to influenza A viruses in poultry, pigs and wild birds varied among months of the year. Although influenza was not detected in samples collected during June, July and August, influenza A virus was detected among samples collected throughout the rest of the year by PCR (Figure 1). Months of highest detection were March and November. Other months with positives but of lower numbers were January, September and December. A similar trend of positive results was observed with ELISA results. Whereas no antibodies were detected in six of the 12 months of the year, antibodies against these viruses were in almost equal proportions in February, May, September and November (Figure 1). Prevalence by PCR in free-living waterfowl in the dry season was 0.7%, while in the wet season it was 2.2%. Comparatively, PCR prevalence of 0.1% and 0.9% for poultry and 0.0% and 1.4% for swine, were observed in the dry and wet seasons, respectively. Comparatively, the seasonal seroprevalence (antibody prevalence by ELISA) of avian influenza in poultry and swine was 0.5% and 6.0% in the dry season (January – February and June – September), while that of the wet season (March - May and October – December) was 0.1% and 3.9%, respectively.

### Prevalence of influenza A viruses among species, age group and management systems

Based on the sampled populations shown in Table 2, the prevalence and seroprevalence of influenza A virus were computed. In the study, prevalence of influenza A virus in domestic ducks and turkeys was 2.7% (95% CI: 1.97 - 49.4; p < 0.01) and 2.6% (95% CI: 3.76 - 22.4), respectively, compared to 0.4% (7/1,865) in chickens. No influenza A virus was detected in guinea fowl samples (N = 8). In swine, RT-PCR positive samples were 1.4% (7/511), while 1.3% (12/929) samples were positive among free-living waterfowl. The proportional distribution of PCR-positive samples among sampled species and regions are presented in Table 3. Influenza A virus was detected only in adults and apparently healthy poultry and pigs. Of the 33 RT-PCR positive samples from poultry and swine, 18 (54.5%) were from male hosts. Except for swine, in which all seven positive samples were from exotic breeds, all positive samples from chickens, domestic ducks and turkeys were from indigenous breeds (Table 4). All PCR positive samples from domestic birds were from flocks kept under free-range management system, while all swine positive nasal swabs were from pigs kept under intensive management system (Table 4). Among species, swine had most of the antibody positive sera (4.6%, 19/417), while lower proportions of positive samples were for the other species including chickens (0.4%, 7/1,863), domestic ducks (0.3%, 2/629) and turkeys (0.0%, 0/76). Generally, prevalence of influenza A virus was 1.0% (26/2,576) and 1.4% (7/511) for poultry and swine, respectively.

**Table 3:** Regional distribution of RT-PCR positive samples among sampled species

**Species**	**Positive RT-PCR samples among regions**			
	**Central**	**Eastern**	**Northern**	**Western**
Chicken (*N = 1,863*)	-	-	2 (0.1%)	5 (0.3%)
Domestic duck (*N = 629*)	-	14 (2.2%)	-	3 (0.5%)
Turkey (*N = 76*)	-	-	-	2 (2.6%)
Guinea fowl (*N = 8*)	-	-	-	-
Swine (*N = 511*)	-	-	-	7 (1.4%)
Waterfowl (*N = 929*)	12 (1.3%)	-	-	-

**Table 4:** Distribution of positive samples among host and management factors

**Species**	**Age group**			**Sex**		**Breed**		**Health status**		**Management system**	
	***Adult***	***Grower***	***Juvenile***	***Female***	***Male***	***Indigenous***	***Exotic***	***Healthy***	***Sick***	***Free-range***	***Intensive***
Chickens	*7 (0.5%)*	*0 (0.0%)*	*0 (0.0%)*	*5 (0.5%)*	*2 (0.2%)*	*7 (0.4%)*	*0 (0.0%)*	*7 (0.4%)*	*0 (0.0%)*	*7 (0.4%)*	*0 (0.0%)*
Domestic ducks	*17 (4.3%)*	*0 (0.0%)*	*0 (0.0%)*	*7 (1.9%)*	*10 (4.0%)*	*17 (1.1%)*	*0 (0.0%)*	*17 (2.3%)*	*0 (0.0%)*	*17 (2.8%)*	*0 (0.0%)*
Turkeys	*2 (2.9%)*	*0 (0.0%)*	*0 (0.0%)*	*0 (0.0%)*	*2 (3.5%)*	*2 (2.6%)*	*0 (0.0%)*	*2 (2.7%)*	*0 (0.0%)*	*2 (2.6%)*	*0 (0.0%)*
Pigs	*7 (3.5%)*	*0 (0.0%)*	*0 (0.0%)*	*3 (0.9%)*	*4 (2.3%)*	*0 (0.0%)*	*07 (3.1%)*	*7 (1.4%)*	*0 (0.0%)*	*0 (0.0%)*	*7 (3.9%)*

Logistic regression analysis indicated that RT-PCR positive samples were more associated with turkeys (OR = 9.9, 95% CI: 3.76 - 22.4; p < 0.01) and ducks (OR = 9.2, 95% CI: 1.97 - 49.4; p < 0.01) compared to the chicken specie. Although sex was not significantly associated with the test results, male poultry had higher odds for testing positive (OR = 1.6, CI: 0.70 - 3.47) than females. Age group had no effect on influenza A virus detection. Comparatively, there was a significant relationship between species and ELISA assay results, with more positive results observed in domestic ducks (OR = 7.7, 95% CI: 3.28 - 18.1) and turkeys (OR = 8.9, 95% CI: 1.82 - 43.7) compared to the chickens. Additionally, male poultry sex was more associated with ELISA results (OR = 1.4, 95% CI: 0.65 - 3.06) compared to female. Whereas no significant difference existed between the test results of infant age compared to adults, growers had lower odds for testing positive (OR = 0.1, >95% CI: 0.02 - 1.07) compare to adults. While in swine, neither age group, sex nor breed had influence on the influenza A virus sera titre results, positive titres were significantly associated with age group (*χ*^2^ = 12.2, 95% CI = 0.004 - 0.007). No association existed between breed and any of the two laboratory assays.

### Prevalence and distribution of influenza A virus subtypes

Of the total number (45) of RT-PCR positive samples, only 13 were positive for H5 subtype and none was positive to H7. Out of the 13 samples that were positive for H5 sub-type, 11 were from wild water birds and 2 were from chickens. All the H5 positive samples were collected in 2010 and none of the 2011 samples were positive to the sub-type. Results of haemagglutination inhibition test revealed that all 19 ELISA positive samples from swine were H1, yet those from poultry showed no clear subtypes by the same test. Based on this result, seroprevalence of H1 influenza A virus subtype in pigs was 3.7%.

## Discussion

Influenza A viruses have been detected in waterfowl [11,21], domestic birds and pigs in several parts of Africa [4,5]. Different tropical countries in Africa and Asia have also reported prevalence of antibodies against avian influenza viruses in poultry [22,23] and swine [24,25]. Although isolation of influenza A virus from RT-PCR positive samples in this study was not successful, RT-PCR results provide the first evidence of occurrence of these viruses in waterfowl, poultry and swine in Uganda. Previous studies have reported that isolation in embryonated chicken eggs is less sensitive than RT-PCR using primers designed based on the most highly conserved regions of the matrix gene, which is up to 100-fold times more sensitive [17]. Detection of influenza A viruses has in several studies been achieved without concurrent isolation in embryonated eggs and mammalian cell cultures [17], which has mainly been attributed to low titres. In cases of failure of isolation or detection of influenza A viruses by RT-PCR assay, serologic results have been used to provide evidence of presence of the same viruses in animal and bird populations [26]. The PCR and ELISA tests used in the study are highly sensitive and specific tests [17,27] that produce reliable results.

A prevalence of 1.3% of avian influenza reported of free-living waterfowl in our study was expected. It is comparable with prevalence of 0.3% - 3.0% among wild birds in North America and is close to 3.0% - 4.0% in other African countries [11,28]. Since some of the species observed at the different sites of sample collection were those that have been reported to harbour LPAI virus in different parts of Africa [9,11], detecting influenza viruses in a country where such birds reside may not be of surprise. *Anseriformes* (ducks, geese, and swans) and *Charadriiformes* (shorebirds, gulls and terns) have previously been described to have a high prevalence of influenza A viruses [11] and a number of species belonging to the two orders of birds have regularly been sighted in Uganda [29]. During a study by Gaidet and colleagues [14], 6.6% and 2.8% of samples taken from Eurasian and African ducks, respectively, were positive to influenza A viruses. Similarly, 3.8% of the samples from Gulls and 1.3% from Terns were RT-PCR positive for the same viruses [14]. These risky bird species were observed in large numbers at study sites during the time samples that turned positive by RT-PCR were collected. Palaearctic migratory birds were associated with the introduction of the HPAI viruses in the wetlands in Nigeria during November and December 2005 before outbreaks of the HPAI in the country later in 2006. Ducks were particularly hypothesized to have acted as bridge species [30].

Variation in prevalence of influenza A viruses in different regions observed in this study is not uncommon and could probably be associated with migration of wild birds and poultry movement. This observation is comparable to previous studies, which have indicated that the spread of HPAI H5N1 virus from Russia and Kazakhstan to the Black Sea basin was consistent in space; and with the hypothesis that birds in the Anatidae family seeded the virus along their autumn migration routes [31]. In China spatial distribution of H5N1 has been observed and epidemiologically linked to poultry trade and wild bird migrations [32].

Seasonal variation in positive samples observed in our study could be expected. Regardless of the host source of samples, no single positive sample was obtained during the months of May – August. In Uganda, these months match with the period when most of the migrant species are either absent or are in significantly small numbers in the country [10,29]. September –April, during which positive samples were collected, is when resident populations are boosted by the many Palaearctic migrants that spend the northern hemisphere winter in Africa [8]. This also broadly coincides with East Africa’s rainy season. Increase in precipitation, high humidity and low temperature could together influence the seasonal variation in prevalence of these viruses. In Uganda the two rainy seasons are March - May and October - December [33] with amount of rainfall averaging 500 mm - 2200 mm/year. In April and November, during which most positive samples were obtained, are months particularly associated with high amount of rainfall, lower temperatures (19°C - 26°C) and higher relative humidity (65% - 96%). Presence of positive samples during September - April period could easily be associated with seasonal presence of migratory waterbird communities [14] during a weather conditions favourable for survival of influenza A viruses. While low relative humidity of 20%–35% has been reported to be most favorable in transmission of influenza viruses [34], no RT-PCR positive sample was obtained during the dry, hot and low humidity season. This discrepancy may on the contrary re-strengthen the notion that high temperatures of Afro-tropical region decrease the potential survival of influenza viruses in the environment [35]. Although seasonal prevalence cycles of influenza A viruses, especially in wild birds, has previously been observed to be consistent with season [31], it can easily be hypothesized that when migratory waterfowl from Europe are present, the influenza A virus circulation considerably increases irrespective of humidity.

The seasonal prevalence of these viruses observed in waterfowl in the dry (0.7%) and wet season (2.2%) was not very different from that obtained in other studies. In a study by Gaidet and others [14], 3.5% prevalence was observed in a large-scale surveillance of water birds in 12 countries in Africa. Proportions of 15% for ducks and geese and 2% for other species have also been reported in an earlier study [1]. In our study, the seasonal prevalence for these viruses in poultry in the dry (0.3%) and wet season (1.3%) and seroprevalence of 0.5% and 0.1% in the same group of species for each of dry and wet season, respectively, were expected in a country that has never had outbreak of HPAI. Similar and varying levels of seroprevalence due to influenza viruses have been reported in poultry and swine [5].

While one of the earlier studies [36] reported higher prevalence of influenza A viruses among juveniles, our results show more positive samples in adult domestic ducks. Similarly, all our positive samples were solely from adult chickens despite the fact that most species of birds, regardless of ages and breed, are considered equally susceptible to influenza A viruses [37]. In a study by Takemae *et al*. [38] in which influenza A viruses were solely isolated from young pigs (4 to 12 weeks); in this study positive serological results were only in adult pigs. Despite having collected most samples from pigs kept under semi-intensive and free-range type of management system, all positive samples were restricted to pigs on intensive system. Intensive system is where larger numbers of pigs are kept.

Detecting influenza A viruses H5 subtype in wild and domestic birds of Uganda was consistent with results of other studies. Work, in which different influenza A virus subtypes have been detected, including that of Gaidet and others in Mali, Munster and colleagues in North America and Caron with others in Southern Africa have been reported in a review by Fouchier and Munster [11]. H5 influenza A virus subtypes (including the H5N1) have particularly been reported to occur in domestic poultry in Africa between 2006 and 2008 [39]. Our haemagglutination inhibition test results showed that all the 19 ELISA-positive swine samples were positive for H1 sub-type, which was not unexpected [40]. While H5N2 HPAI virus has been known to cause disease outbreaks in poultry [41], the LPAI virus H5N2 has been isolated from apparently healthy chickens during routine surveillance in Taiwan [42]. Occurrence of H5 could however be worse for Africa, if the circulating serotype is one of the HPAI form.

The result that the antibodies against influenza A viruses in poultry sera were not sub-typed was an expected observation. While the IDEXX AI Ab Test™ kit used in the study had a panel for detecting antibody reactivity to subtypes of H1-H14, antigens used for HI test were limited to only H1, H3, H5, H6, H7 and H9. Hemagglutinins are subtype-specific and it is possible that hemagglutination inhibition (HAI) test may miss some particular sub-types not tested for. It is very likely that antibodies in the sera were due to influenza subtypes different from those tested by HAI test.

## Conclusions

This study has demonstrated occurrence of influenza viruses in animals in Uganda. Results have specifically suggested that increase in volumes of migratory waterfowl in the country could be associated with increased prevalence of influenza A viruses in free-living waterfowl and poultry, although this may require further study. Swine and domestic birds, especially domestic ducks, would still be ideal for more intensive and longer (longitudinal) studies in order to clearly understand the epidemiology of this group of viruses in Uganda. This could be combined with wild bird capture for cloacal samples to increase chances of virus isolation for complete characterization of the occurring influenza viruses.

## Competing interests

The authors declare that they have no competing interests (be them financial or non-financial) to declare in relation to this manuscript. Therefore, publication of this manuscript will not in any way cause gain or financial loss or otherwise to any organization/institution.

## Authors’ contributions

HKirunda and DKB were involved in study design, sample collection, laboratory analysis, data analysis and manuscript preparation. BE, AT, JK, TT and SM were involved in sample collection, laboratory analysis and manuscript preparation. DM participated in data analysis and manuscript preparation. EM, JB, LL, HKibuuka, MM, AB and FW participated in study design, data analysis and manuscript preparation. MFD, SK, RJW and RGW took part in study design, laboratory analysis, data analysis and manuscript preparation. All authors read and approved the final manuscript.
